# Quo vadis rheumatologische Versorgung in Deutschland? Neue Fachärztezahlen zum 31.12.2024

**DOI:** 10.1007/s00393-025-01720-1

**Published:** 2025-09-05

**Authors:** Katinka Albrecht, Anja Strangfeld, Johanna Callhoff

**Affiliations:** 1 Programmbereich Epidemiologie und Versorgungsforschung, Deutsches Rheuma-Forschungszentrum (DRFZ) Berlin, Charitéplatz 1, 10117 Berlin, Deutschland; 2 Medizinische Klinik mit Schwerpunkt Rheumatologie und Klinische Immunologie, Charité-Universitätsmedizin Berlin, Berlin, Deutschland

**Keywords:** Rheumatologie, Fachkräfte, Ärztemangel, Geschlechterverteilung, Bedarfsplanung, Rheumatology, Specialist workforce shortage, Shortage of doctors, Sex ratio, Requirement planning

## Abstract

**Einleitung:**

Die Anzahl und der Arbeitsumfang der Fachärzt:innen (FÄ) für Rheumatologie sind für die Versorgung von Patient:innen mit entzündlich-rheumatischen Erkrankungen entscheidend. Wir berichten aktuelle Entwicklungen der FÄ-Zahlen bis 2024.

**Methodik:**

Aus den Ärztestatistiken der Bundesärztekammer (BÄK), dem Bundesarztregister der Kassenärztlichen Bundesvereinigung (KBV) und den Grunddaten der Krankenhäuser werden die Anzahl internistischer FÄ für Rheumatologie nach Altersgruppen, Geschlecht, Tätigkeitsbereich, und -umfang berichtet und auf die Bevölkerungszahlen des Statistischen Bundesamtes hochgerechnet.

**Ergebnisse:**

Zum 31.12.2024 gab es 1161 berufstätige FÄ für Rheumatologie, davon waren 531 (46 %) weiblich und 386 (33 %) ≥ 60 Jahre. Anzahl und Anteil berufstätiger FÄ < 60 Jahre sind von 806 (73 %) in 2020 auf 775 (67 %) in 2024 gesunken. An der vertragsärztlichen Versorgung nahmen 725 FÄ teil. Von diesen waren 301 (42 %) angestellt, eine Steigerung im Vergleich zu 2020 (148 [30 %]). Im Gegensatz dazu ist die Zahl an FÄ mit Zulassung von 392 (57 %) auf 365 (50 %) gesunken. Pro 100.000 Erwachsene in Deutschland gab es 1,7 berufstätige und davon 1,0 vertragsärztlich tätige FÄ mit regionaler Varianz von 0,8 im Saarland bis 1,6 in Brandenburg und Hamburg (Personen, nicht Vollzeitkräfte). Seit 2018 ist die Anzahl an FÄ für Rheumatologie in Krankenhäusern von 360 auf 386 in 2023 gestiegen, die Teilzeitbeschäftigung stieg von 25 % auf 39 %. Die Anzahl stationärer Vollkräfte im Jahresdurchschnitt ist von 340 auf 312 gesunken. Von 2020 bis 2024 wurden insgesamt 303 neue FÄ für Rheumatologie anerkannt, was durchschnittlich 61 FÄ pro Jahr entspricht.

**Schlussfolgerung:**

Der Rückgang der Zahl jüngerer FÄ für Rheumatologie führt bei steigender Teilzeitquote zu einer Abnahme der rheumatologischen Kapazitäten in Deutschland. Mit der aktuellen Weiterbildungsquote kann das altersbedingte Ausscheiden von Rheumatolog:innen nicht kompensiert werden, geschweige denn der Verlust an Vollzeittätigkeit.

## Abstract (en)

**Background:**

The number and scope of work of rheumatology specialists are crucial for the care of patients with inflammatory rheumatic diseases. We report on current developments in numbers of rheumatologists up to 2024.

**Methods:**

The number of internal medicine specialists in rheumatology is reported by age group, gender, field of activity, and scope of activity based on medical statistics from the German Medical Association (BÄK), the Federal Register of Physicians of the National Association of Statutory Health Insurance Physicians (KBV), basic data from hospitals, and extrapolated to the population figures of the Federal Statistical Office.

**Results:**

As of 31 December 2024, there were 1161 practicing rheumatology specialists, of whom 531 (46%) were female and 386 (33%) were aged 60 or older. The number and proportion of rheumatologists under the age of 60 fell from 806 (73%) in 2020 to 775 (67%) in 2024. A total of 725 rheumatologists were accredited to work in outpatient practices for national health insurance patients. Of these, 301 (42%) were employed, an increase compared with 2020 (148 [30%]). In contrast, the number of licensed rheumatologists fell from 392 (57%) to 365 (50%). For every 100,000 adults in Germany, there were 1.7 practicing rheumatologists, 1.0 of whom was accredited to outpatient contracted care, with regional variations ranging from 0.8 in Saarland to 1.6 in Brandenburg and Hamburg (persons, not full-time employees). Since 2018, the number of rheumatologists in hospitals has risen from 360 to 386 in 2023; part-time employment increased from 25% to 39%. The annual average number of full-time inpatient staff has fallen from 340 to 312. Between 2020 and 2024, a total of 303 new rheumatology specialists were recognized, corresponding to an average of 61 specialists per year.

**Conclusion:**

The decline in the number of younger rheumatology specialists, coupled with an increase in part-time work, is leading to a decrease in rheumatology capacity in Germany. The current rate of further training is not sufficient to compensate for the age-related retirement of rheumatologists, let alone the loss of full-time positions.

## Einleitung

Seit vielen Jahren beobachten wir die Entwicklung der Anzahl an Fachärzt:innen (FÄ) für Rheumatologie, die für die Versorgung von Patient:innen mit entzündlich-rheumatischen Erkrankungen zur Verfügung stehen. Zunehmende Veränderungen im Arbeitsumfang führen, neben dem Ausscheiden älterer FÄ aus dem Erwerbsleben, zu einem zusätzlichen Verlust an ärztlicher Arbeitszeit und müssen bei der Bedarfsplanung berücksichtigt werden. Diese Entwicklungen kontinuierlich zu beobachten, ist Aufgabe der Versorgungsforschung, um den Versorgungsbedarf für die kommenden Jahre kalkulieren zu können. Bei rückläufigen Kapazitäten müssen rechtzeitig Maßnahmen zur Gegensteuerung entwickelt und eingefordert werden. Dies ist ein wesentliches Ziel der Memoranden, die von der Deutschen Gesellschaft für Rheumatologie und Klinische Immunologie (DGRh) e. V. erstellt werden. Seit der Veröffentlichung des jüngsten Memorandums [1] wurden von den unterschiedlichen Ärzteregistern neue Zahlen veröffentlicht. Die aktuellsten Zahlen inkludierend, berichten wir Trends in der Anzahl an FÄ unter Berücksichtigung der Alters- und Geschlechterverteilung, der Tätigkeitsbereiche und des Arbeitsumfangs bis einschließlich 2024.

## Methodik

Folgende Datenquellen wurden für den Bericht herangezogen:

*Die Ärztestatistiken der Bundesärztekammer* (BÄK) umfassen die Gesamtheit der Ärzt:innen in Deutschland, die Mitglied in den Landesärztekammern sind, zum 31.12. jedes Jahres [2]. Aus den Ärztestatistiken berichten wir die Anzahl berufstätiger FÄ für Rheumatologie und die Anzahl an rheumatologischen Facharztanerkennungen. Da sich die Schwerpunktbezeichnungen in den Weiterbildungsordnungen über die Jahre verändert haben, haben wir die Anzahl der FÄ mit den Bezeichnungen „Innere Medizin und Rheumatologie“, „Innere Medizin und Schwerpunkt (SP) Rheumatologie“, „Innere Medizin, SP Rheumatologie“ und „Innere Medizin Teilgebiet (TG) Rheumatologie“ addiert, um die Gesamtheit aller FÄ für Rheumatologie abzubilden. Darstellungen über mehrere Jahre und stratifiziert nach Altersgruppen und Geschlecht wurden mithilfe der gestaltbaren Tabellen der Gesundheitsberichterstattung des Bundes erstellt [3].

Im *Bundesarztregister der Kassenärztlichen Bundesvereinigung* (KBV) sind alle an der vertragsärztlichen Versorgung teilnehmenden FÄ zum 31.12. jedes Jahres berücksichtigt, also alle FÄ, die Patient:innen der gesetzlichen Krankenkassen ambulant behandeln dürfen [4]. Dies schließt zugelassene und angestellte FÄ sowie persönlich ermächtigte FÄ ein. Nicht enthalten sind ausschließlich in Krankenhäusern tätige FÄ, Institutsermächtigungen, rein privatärztlich tätige FÄ und Weiterbildungsassistent:innen [5]. Aus dem Bundesarztregister berichten wir die Anzahl an FÄ für Rheumatologie, die an der vertragsärztlichen Versorgung teilnehmen, nach Personen und Tätigkeitsbereich (Zulassung, angestellt in einer Einrichtung oder Praxis, Ermächtigung). Zu den Einrichtungen zählen Medizinische Versorgungszentren, Einrichtungen nach § 402 Abs. 2 SGB V (ehemals § 311 SGB V), KV-Eigeneinrichtungen und kommunale Eigeneinrichtungen. Außerdem berichten wir die Anzahl an FÄ nach dem Bedarfsplanungsgewicht, welches die Teilnahmeform und den -umfang der FÄ an der vertragsärztlichen Versorgung berücksichtigt. Die Zählung nach Bedarfsplanungsgewicht entspricht am ehesten der Zählung von Vollzeitäquivalenten (VZÄ), ist jedoch aufgrund der Besonderheiten des Zulassungsrechts und der Bedarfsplanung nicht damit gleichzusetzen [5]. Ermächtigte FÄ werden bei der Zählung nach dem Bedarfsplanungsgewicht nicht berücksichtigt.

Über das *destatis Portal des Statistischen Bundesamts* wurden Bevölkerungszahlen nach Altersgruppen und Bundesländern zum 31.12.2024 abgerufen [6], um die Anzahl an berufstätigen bzw. vertragsärztlich tätigen FÄ für Rheumatologie pro 100.000 Erwachsene in Deutschland und in den Bundesländern zu berechnen. Von *destatis* wurden auch die Zahlen Studierender der Humanmedizin nach Geschlecht für das Jahr 2023 abgerufen [7].

Das Statistische Bundesamt veröffentlicht jedes Jahr im Oktober einen Bericht zu den *Grunddaten der Krankenhäuser* [8]. Hierin enthalten sind die Anzahl an FÄ für Rheumatologie, die in Krankenhäusern tätig sind, nach Geschlecht, Teilzeitbeschäftigung, Anzahl der Vollkräfte im Jahresdurchschnitt und nach Funktion (Anzahl leitend, oberärztlich, assistenzärztlich und belegärztlich tätig). Die Gesamtzahl der Vollkräfte im Jahresdurchschnitt ergibt sich aus der Summe der umgerechneten Teilzeitkräfte, der umgerechneten kurzfristig oder geringfügig beschäftigten Arbeitnehmenden und der Beschäftigten, die im gesamten Jahr bei voller tariflicher Arbeitszeit eingesetzt waren. Der jüngste Bericht umfasst Zahlen aus dem Jahr 2023.

## Ergebnisse

### Berufstätige FÄ für Rheumatologie

**Abb. 1:**
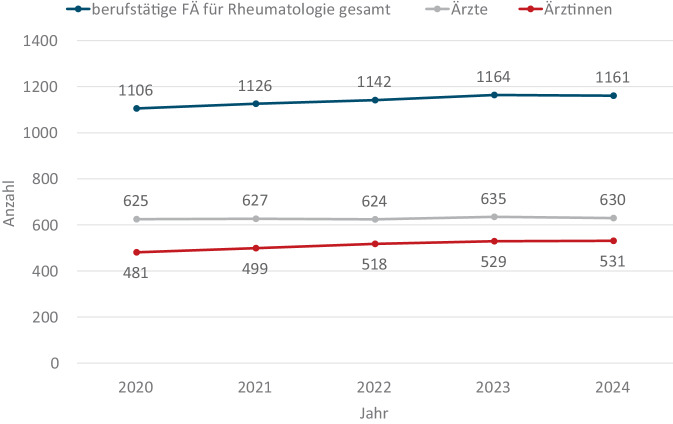
Anzahl berufstätiger FÄ für Rheumatologie nach Geschlecht, Quelle: Eigene Abbildung mit Daten aus Gesundheitsberichterstattung des Bundes https://www.gbe-bund.de/ gestaltbare Tabellen: Ärztinnen und Ärzte mit Schwerpunktbezeichnung bzw. Facharztbezeichnung (Summe) mit ärztlicher Tätigkeit nach Altersgruppen, Ärztestatistik Bundesärztekammer [3]. *FÄ* Fachärzt:innen

Zum 31.12.2024 waren in der Bundesärztekammer (BÄK) 1161 berufstätige FÄ für Rheumatologie gemeldet. Die Anzahl ist seit 2020 (1106) um 55 gestiegen. Der Anteil weiblicher FÄ ist seit 2020 von 43 % auf 46 % gestiegen (Abb. 1).

**Abb. 2:**
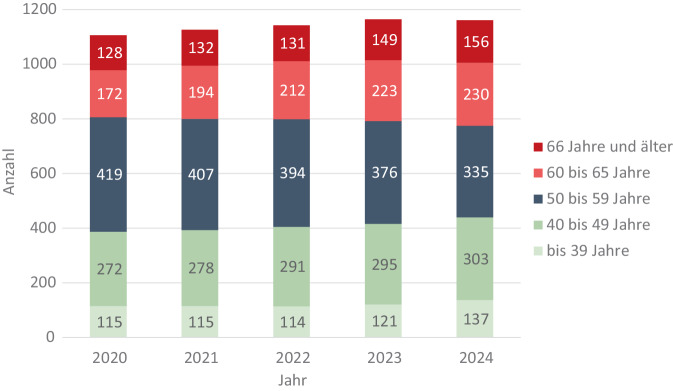
Anzahl berufstätiger Fachärzt:innen für Rheumatologie nach Altersgruppen, Quelle: Eigene Abbildung mit Daten aus Gesundheitsberichterstattung des Bundes https://www.gbe-bund.de/ gestaltbare Tabellen: Ärztinnen und Ärzte mit Schwerpunktbezeichnung bzw. Facharztbezeichnung (Summe) mit ärztlicher Tätigkeit nach Altersgruppen, Ärztestatistik Bundesärztekammer [3]

Im Jahr 2024 waren 386 FÄ 60 Jahre und älter. Der Anteil berufstätiger FÄ ab 60 Jahre ist seit 2020 von 27 % auf 33 % gestiegen. In 2024 gab es zusätzlich 298 FÄ ohne ärztliche Tätigkeit, hiervon waren 237 im Ruhestand oder berufsunfähig. Anzahl und Anteil berufstätiger FÄ < 60 Jahre sind von 806 (73 %) in 2020 auf 775 (67 %) in 2024 gesunken (Abb. 2).

Von den 1161 berufstätigen FÄ für Rheumatologie waren 465 (40 %) stationär und 643 (55 %) ambulant tätig, davon 381 niedergelassen und 262 angestellt.

### In Krankenhäusern tätige FÄ

Die Grunddaten von 2023 listen 143 Krankenhäuser, in denen FÄ für Rheumatologie tätig waren. Insgesamt waren 386 FÄ für Rheumatologie in Krankenhäusern tätig, hiervon waren 176 (46 %) weiblich. Insgesamt waren 149 FÄ (39 %) in Teilzeit bzw. geringfügig beschäftigt. Es gab 72 FÄ in leitender Position, 193 Oberärzt:innen und 121 Assistenzärzt:innen (FÄ) sowie vier Belegärzt:innen. Seit 2018 ist die Anzahl an FÄ für Rheumatologie in Krankenhäusern von 360 auf 386 gestiegen, gleichzeitig stieg der Anteil teilzeitbeschäftigter FÄ von 25 % auf 39 %. Die Anzahl an Vollkräften im Jahresdurchschnitt ist von 340 auf 312 gesunken. Die Anzahl oberärztlich tätiger FÄ hat zu-, die der Belegärzt:innen abgenommen (Tab. 1).

**Tab. 1:** In Krankenhäusern tätige FÄ für Rheumatologie nach Geschlecht und Funktion

	2018	2019	2020	2021	2022	2023
Anzahl Krankenhäuser mit FÄ für Rheumatologie	131	132	139	143	138	143
Anzahl an FÄ für Rheumatologie in Krankenhäusern zum 31.12.	360	361	378	365	371	386
Weiblich, *N* (%)	151 (42 %)	157 (43 %)	165 (44 %)	161 (44 %)	170 (46 %)	176 (46 %)
In Teilzeit/geringfügig beschäftigt, *N* (%)	91 (25 %)	116 (32 %)	126 (33 %)	125 (34 %)	143 (39 %)	149 (39 %)
Leitende Funktion, *N*	73	73	71	69	68	72
Oberärztliche Funktion, *N*	162	167	184	183	193	193
Assistenzärzt:innen (nur FÄ), *N*	125	121	123	121	100	121
Belegärzt:innen, *N*	7	6	8	4	5	4
Vollkräfte im Jahresdurchschnitt	340	322	313	318	306	312

Quelle: Eigene Tabelle mit Daten vom Statistischen Bundesamt (2024) Statistischer Bericht. Grunddaten der Krankenhäuser 2023 [8].

*FÄ* Fachärzt:innen

### Teilnahme an der vertragsärztlichen Versorgung

**Abb. 3:**
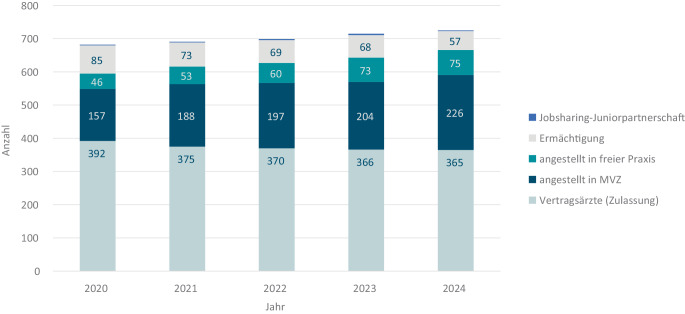
Anzahl an FÄ für Rheumatologie, die an der vertragsärztlichen Versorgung teilnehmen, Quelle: Eigene Abbildung mit Daten aus den Statistischen Informationen aus dem Bundesarztregister zur vertragsärztlichen Versorgung 2020–2024 der Kassenärztlichen Bundesvereinigung (KBV). Stand 31.12. jeden Jahres, Zählung nach Personen, S. 7: FA/SP Rheumatologie [4]. *FÄ* Fachärzt:innen, *MVZ* Medizinisches Versorgungszentrum

Zum 31.12.2024 nahmen 725 FÄ für Rheumatologie an der vertragsärztlichen Versorgung teil. Hiervon hatten 365 eine Zulassung (Vertragsärzte), 301 (42 %) waren in einer Einrichtung oder Praxis angestellt und 57 ermächtigt. Insgesamt 315 FÄ waren Frauen (43 %). Anzahl und Anteil angestellter FÄ sind von 148 (30 %) in 2020 auf 301 (42 %) in 2024 gestiegen, während die Zahl an FÄ mit Zulassung von 392 (57 %) auf 365 (50 %) gesunken ist. Die Zahl ermächtigter FÄ ist von 85 auf 57 zurückgegangen (Abb. 3).

Nach dem Bedarfsplanungsgewicht wurden 530 Personen als Vollzeitäquivalent beschäftigte FÄ für Rheumatologie gezählt, die an der vertragsärztlichen Versorgung teilnehmen (Tab. 2).

**Tab. 2:** An der vertragsärztlichen Versorgung teilnehmende FÄ für Rheumatologie zum 31.12.2024

	Teilnehmende Ärzte	Vertragsärzte	Partnerärzte	Angestellt in Einrichtungen (MVZ)	Angestellt in freier Praxis	Ermächtigte
FA/SP Innere Medizin, Rheumatologie., Zählung nach Personen	725	365	2	226	75	57
Davon weiblich	315	133	1	108	50	23
Zählung nach Bedarfsplanungsgewicht	530	336	–	147	47	–

Quelle: Eigene Tabelle mit Daten aus Gesundheitsberichterstattung des Bundes https://www.gbe-bund.de/ gestaltbare Tabellen: Ärztinnen und Ärzte, die mit einer Schwerpunktbezeichnung bzw. einer bestimmten Facharztbezeichnung an der vertragsärztlichen Versorgung teilnehmen (Anzahl) nach Geschlecht und Teilnahmestatus im Jahr 2024, Bundesarztregister, Kassenärztliche Bundesvereinigung [4]. Zu Einrichtungen zählen Medizinische Versorgungszentren, Einrichtungen nach § 402 Abs. 2 SGB V (ehemals § 311 SGB V), KV-Eigeneinrichtungen und kommunale Eigeneinrichtungen.

*FA* Facharzt, *SP* Schwerpunkt, *FÄ* Fachärzt:innen, *MVZ* Medizinisches Versorgungszentrum

### Regionale Verteilung

Die meisten FÄ für Rheumatologie sind in Bayern und Nordrhein-Westfalen tätig. Bezogen auf die Bevölkerungszahl liegt die Anzahl berufstätiger FÄ für Rheumatologie (Personen, nicht Vollzeitkräfte) zwischen 0,8 (Saarland) und 2,5 (Berlin), für Deutschland insgesamt bei 1,7 pro 100.000 Erwachsene. In Bezug auf die Teilnahme an der vertragsärztlichen Versorgung liegt die Anzahl zwischen 0,8 (Saarland) und 1,6 (Brandenburg, Hamburg), für Deutschland insgesamt bei 1,0 pro 100.000 Erwachsene (Tab. 3).

**Tab. 3:** Regionale Verteilung berufstätiger und vertragsärztlich tätiger FÄ für Rheumatologie zum 31.12.2024

	Erwachsene Bevölkerung	Berufstätige FÄ für Rheumatologie		An der vertragsärztlichen Versorgung teilnehmende FÄ für Rheumatologie	
		Anzahl	Pro 100.000 Erwachsene	Anzahl	Pro 100.000 Erwachsene
Deutschland	69.602.098	1161	1,7	725	1,0
Baden-Württemberg	9.300.631	146	1,6	80	0,9
Bayern	11.013.635	229	2,1	113	1,0
Berlin	3.075.666	77	2,5	37	1,2
Brandenburg	2.145.650	34	1,6	34	1,6
Bremen	582.755	9	1,5	4	0,7
Hamburg	1.546.327	25	1,6	25	1,6
Hessen	5.213.228	94	1,8	49	0,9
Mecklenburg-Vorpommern	1.333.656	26	1,9	17	1,3
Niedersachsen	6.655.972	105	1,6	71	1,1
Nordrhein-Westfalen	14.970.948	206	1,4	144	1,0
Rheinland-Pfalz	3.437.893	49	1,4	23	0,7
Saarland	855.981	7	0,8	7	0,8
Sachsen	3.397.437	64	1,9	47	1,4
Sachsen-Anhalt	1.816.303	33	1,8	25	1,4
Schleswig-Holstein	2.477.082	40	1,6	30	1,2
Thüringen	1.778.934	17	1,0	19	1,1

Quellen: Eigene Tabelle mit Daten aus Bevölkerung nach Bundesländern und Altersjahren zum 31.12.2024, https://www-genesis.destatis.de/datenbank/online/statistic/12411/table/12411-0012, Gesundheitsberichterstattung des Bundes https://www.gbe-bund.de/ gestaltbare Tabellen: Ärztinnen und Ärzte mit Schwerpunktbezeichnung bzw. Facharztbezeichnung (Summe) mit ärztlicher Tätigkeit nach Region in 2024, Ärztestatistik der Bundesärztekammer; Ärztinnen und Ärzte, die mit einer Schwerpunktbezeichnung bzw. einer bestimmten Facharztbezeichnung an der vertragsärztlichen Versorgung teilnehmen (Anzahl) nach Region im Jahr 2024, Bundesarztregister, Kassenärztliche Bundesvereinigung.

*FÄ* Fachärzt:innen

### Facharztanerkennungen

Im Jahr 2024 wurden 66 FÄ für Innere Medizin und Rheumatologie bzw. Schwerpunkt Rheumatologie anerkannt. Von 2020 bis 2024 wurden insgesamt 303 neue FÄ, d. h. im Durchschnitt 61 FÄ pro Jahr, anerkannt. Der Anteil an Frauen lag zwischen 55 und 68 % (Tab. 4).

**Tab. 4:** Anzahl an Facharztanerkennungen für Rheumatologie 2020–2024

	2020	2021	2022	2023	2024
FA/SP Innere Medizin und Rheumatologie	63	60	58	56	66
Davon Frauen	43 (68 %)	33 (55 %)	35 (60 %)	31 (55 %)	40 (61 %)

Quelle: Daten aus Ärztestatistiken der Bundesärztekammer von 2020–2024, Tab. 9, S. 36 [2].

*FA* Facharzt, *SP* Schwerpunkt

**Abb. 4:**
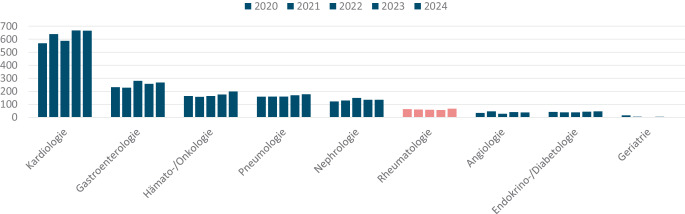
Anzahl an Facharztanerkennungen 2020–2024 in den Teilgebieten der Inneren Medizin, Quelle: Eigene Abbildung, Daten aus Ärztestatistiken der Bundesärztekammer von 2020–2024, Tab. 9, S. 36 [2] , Addition von Schwerpunkt und Facharztbezeichnungen für die jeweiligen Fachbereiche

Im Vergleich zu den anderen Schwerpunkten der Inneren Medizin liegt die Rheumatologie hinsichtlich der jährlichen Facharztanerkennungen im unteren Bereich, sowohl hinsichtlich der Gesamtzahl als auch im Zuwachs in den letzten fünf Jahren (Abb. 4).

#### Geschlechterverhältnis in der Rheumatologie

**Abb. 5:**
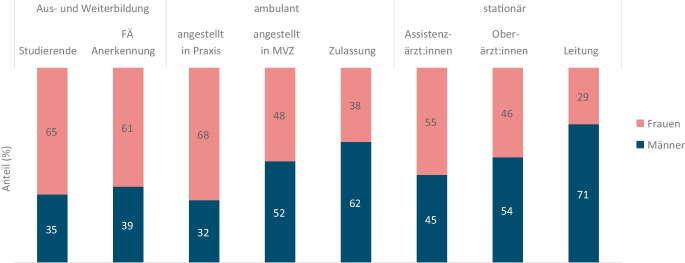
Geschlechterverteilung bei Studierenden und FÄ für Rheumatologie nach Tätigkeitsbereichen, Quellen: Eigene Abbildung mit Daten von: Studierende der Humanmedizin 2023 nach Geschlecht, destatis [7]; Facharztanerkennungen 2020–2024, Ärztestatistiken der Bundesärztekammer [2], Bundesarztregister 2024 der Kassenärztlichen Bundesvereinigung [4] und Grunddaten der Krankenhäuser 2023 [8]. *FÄ* Fachärzt:innen, *MVZ* Medizinisches Versorgungszentrum

In 2023 waren von 113.383 Studierenden der Humanmedizin 73.244 (65 %) Frauen. Bei den Facharztanerkennungen für Rheumatologie lag der Anteil an Frauen von 2020 bis 2024 im Durchschnitt bei 61 %. In der vertragsärztlichen Versorgung waren von den in freier Praxis angestellten FÄ für Rheumatologie 68 % Frauen, von den angestellten FÄ in Einrichtungen wie Medizinischen Versorgungszentren waren dies 48 % und von den Vertragsärzt:innen mit einer Zulassung 38 %. In den Krankenhäusern waren 55 % der assistenzärztlich tätigen FÄ weiblich, 46 % der oberärztlich tätigen FÄ und 29 % der leitenden FÄ (Abb. 5).

## Diskussion

Seit mehr als fünf Jahren weisen Daten aus der Versorgungsforschung wiederholt auf den drohenden Mangel rheumatologischer FÄ hin [1, 9–12]. Zahlreiche Appelle an politisch Verantwortliche und eine groß angelegte Öffentlichkeitskampagne „rheuma2025.de“ des Bündnisses für Rheumatologie wurden an medizinisch ausbildenden Universitäten und in verschiedenen Medienkanälen über mehrere Jahre durchgeführt [13]. Viel geändert hat sich an den rheumatologischen FÄ-Kapazitäten seitdem nicht. Der bereits 2021 adressierte hohe Anteil an rheumatologischen FÄ über 60 Jahre [9] hat weiter zugenommen und 2024 zeigt sich erstmals eine deutliche Abnahme nicht nur im Anteil, sondern auch in der Anzahl an FÄ unter 60 Jahren. Die stark gestiegene Teilzeitbeschäftigung führt sowohl im stationären als auch im ambulanten Bereich zu reduzierten ärztlichen Arbeitszeiten. Der Rückgang zugelassener Vertragsärzt:innen geht mit einem deutlichen Anstieg angestellter FÄ im ambulanten Bereich einher. Der Mindestbedarf von zwei Vollzeitäquivalent tätigen FÄ für Rheumatologie pro 100.000 Erwachsene für die ambulante Versorgung [14] wird bei Weitem nicht gedeckt, selbst wenn alle berufstätigen FÄ für Rheumatologie berücksichtigt werden.

Alle diese Entwicklungen sind nicht spezifisch für die Rheumatologie, sondern betreffen andere medizinische Fachbereiche im In- und auch im Ausland [15, 16]. Allerdings zeigt der Vergleich der Facharztanerkennungen der Inneren Medizin, dass es Unterschiede in der Anzahl und im Zuwachs bei den einzelnen Schwerpunkten gibt. Auch in anderen Ländern gibt es bereits große Schwierigkeiten, ausreichend rheumatologische FÄ zu finden [17, 18], sodass Abwerbungen gut ausgebildeter rheumatologischer FÄ, z. B. in die Schweiz, längst verbreitet sind. Die FÄ gehen dorthin, wo sie die besten Arbeitsbedingungen vorfinden.

Jetzt wird es wirklich kritisch und wir benötigen diverse Strategien, um mehr ärztliche Arbeitszeit zu generieren, aber auch um die bestehende Arbeitszeit effizienter zu nutzen [15]. Wichtig ist zusätzlich, dass wir für die ärztliche Tätigkeit in der rheumatologischen Patientenversorgung Arbeitsbedingungen schaffen, die für die derzeit tätigen FÄ mit einer höheren Zufriedenheit einhergehen, damit wir sie nicht bei steigender Belastung in andere Bereiche mit attraktiveren Arbeitsbedingungen verlieren.

Die Anzahl an Facharztanerkennungen war 2024 mit 66 erfreulicherweise wieder deutlich höher als in den Vorjahren. Ob dies ein Trend ist oder eher eine jährliche Schwankung, wie es sie bereits in früheren Jahren gab, werden die Zahlen der nächsten Jahre zeigen.

Die Rheumatologie wird weiblicher, das zeigt sich in dem Anteil an Studentinnen und Ärztinnen in Weiterbildung sehr deutlich. Auch in einer Umfrage unter Weiterbildungsassistent:innen in der Rheumatologie waren 68/102 (67 %) Frauen [19], Statistiken zu den Ärzt:innen in rheumatologischer Weiterbildung gibt es nicht. Die unterschiedlichen Geschlechterverteilungen in den einzelnen Tätigkeitsbereichen zeigen, dass weibliche FÄ für Rheumatologie sehr viel häufiger im Angestelltenverhältnis in einer Praxis beschäftigt sind, während ihr Anteil an Positionen mit Leitungsfunktion deutlich geringer ist.

## Limitationen der Datenquellen

In der Ärztestatistik der BÄK werden Ärzt:innen von den Landesärztekammern in anonymisierter Form an die BÄK gemeldet. Die Zuordnung der FÄ zu den einzelnen Fachgebieten erfolgt durch die Landesärztekammern. Die Einteilung nach ambulanter und stationärer Tätigkeit beruht auf Eigenangaben der Mitglieder. Um Mehrfachzählungen zu vermeiden, wird der BÄK nur der zuletzt erworbene Facharzttitel gemeldet, weitere Facharzttitel werden nicht berücksichtigt. Im Bundesarztregister wird ein FA, wenn er mehr als einen Schwerpunkt hat, mehrfach gezählt. Aufgrund der unterschiedlichen Erhebungen können gewisse Abweichungen zwischen den Daten der Ärztestatistik, dem Bundesarztregister und den Grunddaten der Krankenhäuser vorliegen.

## Fazit für die Versorgung

Bei kontinuierlich steigenden Patientenzahlen sinken die für die Versorgung zur Verfügung stehenden fachärztlichen Kapazitäten in der Rheumatologie. Der baldige Renteneintritt der über 60-jährigen FÄ für Rheumatologie und die zunehmende Teilzeittätigkeit der jüngeren FÄ können nicht durch die derzeitige Weiterbildungsquote ausgeglichen werden. Neben der Ausbildung neuer FÄ benötigen wir Strategien zur effizienteren Gestaltung der Versorgungsprozesse sowie Konzepte für Arbeitsbedingungen, die die derzeitigen FÄ für Rheumatologie für unser Fachgebiet erhalten.
